# A survey-based study of factors that motivate nurses to protect the privacy of electronic medical records

**DOI:** 10.1186/s12911-016-0254-y

**Published:** 2016-02-02

**Authors:** Chen-Chung Ma, Kuang-Ming Kuo, Judith W. Alexander

**Affiliations:** 1 Department of Healthcare Administration, I-Shou University, No.8, Yida Rd., Yanchao District, Kaohsiung City, ROC 82445 Taiwan; 2 University of South Carolina, 4389 Oakman St S., Salem, OR USA

**Keywords:** Decomposed theory of planned behavior, Electronic medical records, Privacy protection, Nurses

## Abstract

**Background:**

The purpose of this study is to investigate factors that motivate nurses to protect privacy in electronic medical records, based on the Decomposed Theory of Planned Behavior.

**Methods:**

This cross-sectional study used questionnaires to collect data from nurses in a large tertiary care military hospital in Taiwan.

**Results:**

The three hundred two (302) valid questionnaires returned resulted in a response rate of 63.7 %. Structural equation modeling identified that the factors of attitude, subjective norm, and perceived behavioral control of the nurses significantly predicted the nurses’ intention to protect the privacy of electronic medical records. Further, perceived usefulness and compatibility, peer and superior influence, self-efficacy and facilitating conditions, respectively predicted these three factors.

**Conclusions:**

The results of our study may provide valuable information for education and practice in predicting nurses’ intention to protect privacy of electronic medical records.

## Background

International advocacy has grown considerably to use healthcare information technologies (HITs), such as electronic medical records (EMRs), to enhance healthcare service quality and decrease costs [1–3]. Despite expected clinical and economic benefits for EMRs, the privacy of health care data remains a concern for both patients and healthcare organizations, impacting the use of EMRs [4].

Nursing plays an important role in patient care services since health care organizations recognize nurses as both coordinators and providers of these services [5]. Nurses comprise the largest portion of healthcare professionals and interact more with EMRs than other health care professionals due to the nature of their work [6]. The adoption of EMRs should assist nurses in providing nursing care and completing record-keeping routines more efficiently and effectively. Clinically, nurses collect and disseminate confidential patient information as part of their daily routines [7]. Consequently, besides caring for patients, the role of the nurse includes protecting the personal information of patients [7] as stated in the *Code of Ethics for Nurses* from the American Nurses Association [8] and the Nightingale Pledge [9]. Further, the privacy rules of Health Insurance Portability and Accountability Act (HIPAA) require healthcare providers to secure the privacy of patients’ health information [10]. Failure of nurses to safeguard patient privacy will erode nurse/patient relationships and impact the quality of the treatment provided [11]. Further, if nurses do not maintain the privacy of the information in EMRs, thus inappropriately disclose such information; patients may receive serious harm [12]. Unfortunately, most violations of patient privacy in medical facilities result from staff abuse or misuse of the right to access patient records [13].

Previous nursing studies regarding privacy mainly focused on providing opinion reports describing the importance of protecting patient privacy [7, 11]. However, little research has explored empirically the factors influencing nurses to protect the privacy of EMRs. By knowing these influencing factors, medical facilities can formulate strategies to motivate nurses’ intention to protect the privacy of EMRs. Consequently, the primary purpose of our study is to explore empirically the factors motivating nurses to protect EMRs privacy based on the Decomposed Theory of Planned Behavior (DTPB) [14].

### Literature review

#### Electronic medical records

Generally, managers consider Information Technologies (ITs) to be an effective tool for improving efficiency and effectiveness in organizations. Although the healthcare industry has lagged behind other industries in the utilization of ITs, many medical facilities have understood the benefits that ITs offer and have adopted ITs [1]. The EMR is one type of IT that healthcare managers expect will reduce cost and improve the quality of health care [1, 2]. EMRs refer to the array of computer software applications commonly used to communicate orders for medical care, to document pertinent facts regarding a patient’s medical history, and to disseminate results of diagnostics testing [4].

Health care professionals often use another term, electronic health records (EHRs), interchangeably with EMRs. However, these two terms are different in several ways. First, EMRs are legal records created in hospitals and are the source of EHRs [15]. EMRs primarily contain the medical and treatment history of the patients in one hospital and only used by healthcare professionals within that hospital [16]. On the other hand, EHRs also contain wellness information [15, 16]. Thus, EHRs provide a broader view on a patient’s care than EMRs.

Additionally, health care professionals have used the concept of personal health records (PHRs) to preserve patient data. PHRs refer to “*an electronic application through which individuals can access*, *manage and share their health information*, *and that of others for whom they are authorized*, *in a private*, *secure*, *and confidential environment*” ([17], p. 122). PHRs gather health data entered by individuals and can provide individuals’ health information to healthcare professionals under authorizations by those individuals [18]. Further, PHRs can also capture data from EMRs to share over many hospitals, since patients could receive care from different hospitals [17]. Thus, our study adopts the term EMRs, since we focus on the electronic medical record in one hospital where most of the information is medical data gathered at that hospital, rather than data related to wellness and data gathered across institutions.

International proponents in the healthcare industry consider the wide-scale adoption of EMRs as essential [1, 2]. In 2012, 44 % of U.S. healthcare practitioners used some kind of EMRs [19]. In Taiwan, about 65.2 % of hospitals have adopted EMRs [20]. The rate of EMRs adoption in Japan in 2011 was 51.5 % in large hospitals [21]. In their study of EMRs adoption in China, Shu et al. [22] report that about 69.3 % of hospitals have their physicians using EMRs to place orders. An estimate is that by 2020, approximately 50 % of practitioners in USA will be using functional EMRs [1]. Accenture [23] even predicted that global EMRs market would reach $22.3 billion (USD) by the end of 2015. Thus, the study of EMRs is important.

#### Privacy

Privacy is an individual’s right to determine which personal information to share with whom and for what purposes [11]. The invasion of privacy occurs when individuals cannot control the disclosure and usage of their personal information [24]. In healthcare settings, privacy refers to the ability of individuals to prevent certain disclosure of personal health information to others [25]. In addition to personal information such as the basic health information of height and blood pressures, the medical records may also include the more sensitive personal information such as sexually transmitted diseases, abortions, emotional problems, and physical abuse [24, 25]. Researchers have found that individuals have increasing concerns pertaining to whether organizations (including medical facilities) are proficient in safeguarding their personal information [24] including health-related information [26]. With the increasing usage of EMRs, more personal health information is stored and even shared among medical facilities and their staffs, which places the privacy of patients’ personal health information at a greater risk [7].

#### Research framework and hypotheses development

Among the well-known intention-behavior models, scientists have widely adopted the Theory of Planned Behavior (TPB) [27] to predict an individual’s behavior in numerous settings including healthcare-related settings [28–30]. The TPB postulates that an individual’s behavioral intention is a function of attitude, subjective norm, and perceived behavioral control. *Attitude* refers to the positive/negative evaluations by an individual toward performing a behavior [28]. *Subjective norm* means the perceptions that significant referents desire the individual to perform or not perform a behavior [14]. *Perceived behavioral control* refers to the perceptions of internal and external constraints on behavior [27]. TPB [27] has been widely adopted to predict individual’s behavior in various disciplines. However, Taylor and Todd [14] argue that to better understand the relationships between multiple beliefs and the three antecedents of intention (i.e., attitude, subjective norm, and perceived behavioral control), further decomposition of attitudinal beliefs is required. Since their seminal work on the Decomposed Theory of Planned Behavior [14], numerous studies have adopted the model to predict individuals’ intention toward a specific behavior. Previous research [28, 31–33] on individuals’ attitudes towards HITs has demonstrated DTPB performs better than TPB.

**Fig. 1:**
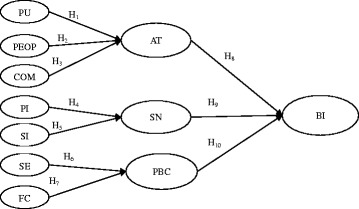
Research framework (Adapted with permission. Copyright 1995 INFORMS. Shirley Taylor, Peter A. Todd (1995) Understanding Information Technology Usage: A Test of Competing Models. *Information Systems Research* 6(2):144–176, the Institute for Operations Research and the Management Sciences, 5521 Research Park Drive, Suite 200, Catonsville, Maryland 21228, USA). Note: PU (perceived usefulness), PEOP (perceived ease of protection), COM (compatibility), PI (peer influence), SI (superior influence), SE (self-efficacy), FC (facilitating conditions), AT (attitude), SN (subjective norm), PBC (perceived behavioral control), BI (behavioral intention)

Our study adopted the DTPB [14] as the research framework to investigate nurses’ behavioral intentions to protect the privacy of EMRs. As Fig. 1 shows, the three primary antecedents (i.e., attitude, subjective norm, and perceived behavioral control) directly influence behavioral intentions can be decomposed into multidimensional constructs. Perceived usefulness (PU), perceived ease of protection (PEOP), and compatibility (COM) influence attitude (AT). Further, peer influence (PI) and superior influence (SI) collectively influence subjective norm (SN). In addition, self-efficacy (SE) and facilitating conditions (FC) influence perceived behavioral control (PBC). Figure 1 shows the justification of the research framework and the research constructs and their associations.

#### Effects of perceived usefulness, perceived ease of protection, and compatibility on attitude

According to DTPB [14] as we use in this study, attitude refers to the extent that a nurse holds a favorable or unfavorable evaluation of protecting the privacy of EMRs [27]. Taylor and Todd [14] decompose attitude into three constructs (i.e., perceived usefulness, perceived ease of use, and compatibility) to predict users’ intentions to adopt new ITs. In our study setting, perceived usefulness depicts that protecting the privacy of EMRs is beneficial to nurses and hospitals. Perceived ease of use, originally defined as a person’s belief that using a particular system would be free of effort [34], is not suitable in our study context since we aim to investigate nurses’ intentions to protect the privacy of EMRs, not to adopt an IT. Consequently, we use ‘perceived ease of protection,’ which refers to the degree that nurses believe protecting the privacy of EMRs would be free of effort, thus corresponding to the ‘perceived ease of use.’ While compatibility refers to protecting the privacy of EMRs when nurses perceive the protection is consistent with existing values, needs, and experiences of nurses. Previous studies [14, 28, 31, 35–37] affirm that perceived ease of use (protection), perceived usefulness, and compatibility significantly influence individuals’ attitude towards new ITs. Accordingly, the following hypotheses are proposed:

- H_1_: Perceived usefulness has a positive influence on nurses’ attitudes toward protecting privacy of EMRs.
- H_2_: Perceived ease of protection has a positive influence on nurses’ attitudes toward protecting privacy of EMRs.
- H_3_: Compatibility has a positive influence on nurses’ attitudes toward protecting privacy of EMRs.

#### Effects of peer influence and superior influence on subjective norm

Subjective norm is a type of subjective social pressure derived from a similar group of people (e.g., friends, colleagues, or superiors), influencing an individual’s attitude toward his/her intentions [27]. Individuals are more likely to comply with others’ expectations when those referent others have the ability to reward the desired behavior or punish non-behavior [38]. That is, peer behaviors may play an important role in motivating individuals to perform a specific behavior. Taylor and Todd [14] also confirm that the influences of both peers and superiors have direct effects on subjective norms. Consequently, a reasonable supposition is that peers or superiors would influence nurses’ perceived subjective norm. This reasoning leads to the following hypotheses:

- H_4_: Peer influence has a positive influence on nurses’ subjective norms toward protecting privacy of EMRs
- H_5_: Superior influence has a positive influence on nurses’ subjective norms toward protecting privacy of EMRs

#### Effects of self-efficacy and facilitating conditions on perceived behavioral control

Based on prior studies [14, 38], perceived behavioral control refers to the internal and external constraints on protecting privacy of EMRs and in our study includes self-efficacy and facilitating conditions. Self-efficacy refers to the self-confidence of nurses in their ability to protect the privacy of EMRs, while facilitating conditions refers to resources such as EMRs related hardware, software, and usability in expediting privacy-protection behaviors that are compatible with existing hardware and software in hospitals. Extant literature [27, 28] confirms that the self-efficacy and facilitating conditions affect an individual’s perceived behavioral control. Therefore, the following hypotheses are proposed:

- H_6_: Self-efficacy has a positive influence on nurses’ perceived behavioral control toward protecting privacy of EMRs
- H_7_: Facilitating conditions have a positive influence on nurses’ perceived behavioral control toward protecting privacy of EMRs

#### Effects of attitude, subjective norm, and perceived behavioral control on behavioral intention

Based on TPB [27], three primary constructs including attitude, subjective norm, and perceived behavioral control predict an individual’s behavioral intention. Various literature [14, 28, 30] also suggests that these relationships exist. Transferring the rationale of TPB into our study, we suggest that if nurses hold positive attitudes toward privacy measures proposed by the hospital, they will be more willing to engage in EMRs privacy protection behavior. Moreover, individuals’ perceived social pressure from people they care about usually can influence them to perform a given behavioral action [27]. If nurses believe that their colleagues, superiors, or even friends expect protection of patient privacy, they will be more likely to protect the privacy of EMRs. Finally, individuals usually assess whether they have the requisite resources to overcome obstacles encountered to perform a specific behavior [27]. Consequently, nurses who feel capable of protecting patient privacy are more willing to engage in privacy-protecting activities related to EMRs. In light of above discussions, we propose the following hypotheses:

- H_8_: Nurses’ attitudes have a positive influence on their behavioral intention to protect privacy of EMRs
- H_9_: Nurses’ subjective norms have a positive influence on their behavioral intention to protect privacy of EMRs
- H_10_: Nurses’ perceived behavioral controls have a positive influence on their behavioral intention to protect privacy of EMRs

## Methods

### Design

To assess the perceptions of nurses regarding privacy protection of EMRs, we undertook a cross-sectional survey at a tertiary care military hospital in Taiwan. The hospital, with 732 beds, provides tertiary care service to both military and civilians patients resulting in more than 19,860 annual patient admissions in 2013. The hospital adopted EMRs in 2009, with all nurses documenting care records in EMRs. This fact indicates that the nurses should have adequate knowledge concerning the operations of EMRs to participate in this study.

### Instrument development

The constructs in our research framework were measured using 32 items from previous validated works [14, 38]. An expert panel consisting of one senior hospital manager and two experienced researchers in the field of healthcare information management inspected these items. The panel considered one (1) of the items redundant and suggested removal of this item; while the researchers modified other items based on the recommendations from the experts (See Appendix 1 for the removed item). We used a 7-point Likert scale (1 for ‘strongly disagree’ and 7 for ‘strongly agree’) to assess the survey items since a 7-point scale is currently the most widely used type of scale [39] and is more reliable than a 5-point scale [40]. Further, a 7-point scales can prevent people from being too neutral in their responses [41] and is comparable with a 5-point scale [42]. Table 1 depicts the final measurement items for constructs of interest and their sources.

**Table 1:** Constructs of interest and corresponding items

Constructs (abbreviation)	Items	Measure
Perceived usefulness (PU) [14]	PU1	Protecting EMRs privacy is beneficial to me
	PU2	The advantages of protecting EMRs privacy outweigh the disadvantages
	PU3	Protecting EMRs privacy will improve patients’ trust on hospitals
Perceived ease of protection (PEOP) [14]	PEOP1	The instructions for protecting EMRs privacy is easy to follow
	PEOP2	It is easy to learn how to protect EMRs privacy
	PEOP3	It is easy to protect EMRs privacy
Compatibility (COM) [38]	COM1	Protecting EMRs privacy fits into my work style
	COM2	I think that protecting EMRs privacy fits well with the way I like to work
	COM3	Protecting EMRs privacy is compatible with all aspects of my work
Peer influence (PI) [14]	PI1	My friends would think that I should protect EMRs privacy
	PI2	My colleagues would think that I should protect EMRs privacy
Superior influence (SI) [14]	SI1	My superior would think that I should protect EMRs privacy
	SI2	I will protect EMRs privacy because my superior asks for
Self-efficacy (SE) [14]	SE1	I could easily protect EMRs privacy if I wanted to
	SE2	I could protect EMRs privacy if there was no one around to tell me what to do as I go
	SE3	I would feel comfortable in protecting EMRs privacy
Facilitating conditions (FC) [14]	FC1	The equipment (computers, printers, etc.) for EMR systems is compatible with other hardware I use in hospital
	FC2	The software for EMR systems is compatible with other software I use in hospital
	FC3	I could use EMR systems to query patient’s medical records
Attitude (ATT) [38]	ATT1	Protecting EMRs privacy is a good idea
	ATT2	I think protecting EMRs privacy is a wise idea
	ATT3	I like the idea of protecting EMRs privacy
	ATT4	Protecting EMRs privacy is fun
Subjective norm (SN) [38]	SN1	People who influence my behavior would think that I should protect EMRs privacy
	SN2	People who are important to me would think that I should protect EMRs privacy
Perceived behavioral control (PBC) [38]	PBC1	I would be able to protect EMRs privacy
	PBC2	I have the knowledge necessary to protect EMRs privacy
	PBC3	I have the resources necessary to protect EMRs privacy
Behavioral intention (BI) [38]	BI1	I intend to protect EMRs privacy
	BI2	I predict I would protect EMRs privacy
	BI3	I plan to protect EMRs privacy

### Survey procedure and ethics approval

We used a field survey to test the proposed model. We obtained approval from the Institutional Review Board (IRB) of Kaohsiung Armed Forces General Hospital prior to proceeding with the investigation. The IRB waived the mandate for obtaining informed consent from subjects. We distributed questionnaires to all of the 474 registered nurses in the subject hospital. In December 2012, nurses, voluntarily and anonymously, completed the paper-and-pencil survey. In all, we collected 307 responses, indicating a response rate of 63.7 %. We had 302 responses for analysis since we eliminated five questionnaires because of partial answers.

### Common method bias

Regarding common method bias, we used the Harman’s single factor test [43] to check whether significant method effects occurred on our hypothesized relationships. We use confirmatory factor analysis (CFA) to detect this issue as suggested by literature [43]. All the manifested items were modeled as the indicators of a single factor and the CFA results revealed poor fit between the collected data and the model (e.g., *χ*2/d.f. = 9.97; CFI = .74; RMSEA = .17). Common method bias should not be a problem in our study.

## Results

### Descriptive statistics

Of the 302 valid responses, 296 were female (98 %) and six (6) were male (2 %). Nearly 75 % of the respondents were 30-49 years of age. In addition, the majority of respondents (99.3 %) were college- or university-educated. Further, about 8.6 % of the respondents were managerial level staff. All of the respondents had experiences in using EMRs in the subject hospital, indicating these respondents should have had adequate background knowledge about the survey content to render a meaningful response. Table 2 shows the respondents’ demographics.

**Table 2:** Respondent characteristics

Variable	Category	Frequency	Percentage
Gender	Male	6	2
	Female	296	98
Age (years)	18–29	58	19.2
	30–49	226	74.8
	50–64	18	6
Education level	High school	2	7
	College	281	93
	University	19	6.3
Working position	Non-managerial	276	91.4
	Managerial	26	8.6

### Data analysis

We empirically validated the proposed model using partial least squares (PLS), supported by SmartPLS® 2.0 M3 software [44]. PLS (a variance-based structural equation model) reduces the effect of measurement error by creating a weighted sum from multiple indicators of a latent variable to account for measurement error. As such, PLS handles measurement error differs from covariance-based structural equation, which explicitly includes measurement error in the research model [45]. Currently, researchers have no clear census concerning whether measurement error should be modeled or eliminated [46]. We chose PLS for its ability to handle latent constructs with non-normality and with small to medium sample sizes [47].

### Measurement model

We first assessed the measurement model according to three tests: reliability, convergent validity, and discriminant validity [47]. Reliability can be gauged via factor loading, composite reliability (CR), and Cronbach’s α [47]. The factor loadings of all constructs exceeded the suggested criterion of .7 [48], demonstrating adequate item reliability (see Table 3). In addition, the figures of CR and Cronbach’s α scores were higher than the recommended .7 thresholds, indicating acceptable reliability. Regarding convergent validity, the value of average variance extracted (AVE) exceeded .5 implying convergent validity [48] (see Table 3). Meanwhile, the inter-construct correlations matrix (see Table 4) demonstrates that the square root of AVE for each construct exceeded the correlation of the specific construct with any other constructs in the model, thus indicating sufficient discriminant validity [48].

**Table 3:** Descriptive statistics and reliability measures

Construct	Items	Mean	SD	Loadings	AVE	CR	Cronbach’s α
PU	PU1	6.09	0.94	.97	.92	.97	.96
	PU2	6.01	0.97	.96			
	PU3	6.07	0.95	.95			
PEOP	PEOP1	5.68	1.03	.95	.91	.97	.95
	PEOP2	5.68	1.03	.95			
	PEOP3	5.64	1.04	.96			
COM	COM1	5.77	0.94	.98	.96	.98	.98
	COM2	5.75	0.94	.98			
	COM3	5.76	0.93	.98			
PI	PI1	5.80	1.01	.97	.95	.98	.95
	PI2	5.89	0.98	.98			
SI	SI1	6.05	0.93	.97	.93	.96	.93
	SI2	5.97	1.00	.96			
SE	SE1	5.86	0.96	.94	.91	.97	.95
	SE2	5.70	1.12	.95			
	SE3	5.75	1.02	.96			
FC	FC1	5.60	1.06	.95	.91	.97	.95
	FC2	5.62	1.07	.97			
	FC3	5.59	1.05	.94			
ATT	ATT1	6.00	0.90	.93	.88	.97	.96
	ATT2	5.88	0.94	.96			
	ATT3	5.87	0.95	.96			
	ATT4	5.72	1.05	.90			
SN	SN1	5.90	0.96	.99	.98	.99	.82
	SN2	5.89	0.99	.99			
PBC	PBC1	5.88	0.92	.96	.93	.98	.96
	PBC2	5.81	1.00	.97			
	PBC3	5.75	0.99	.96			
BI	BI1	5.92	0.96	.97	.92	.97	.96
	BI2	5.88	0.97	.96			
	BI3	5.97	0.92	.94			

CR denotes composite reliability, AVE denotes average variance extracted

**Table 4:** Inter-construct correlations

	PU	PEOP	COM	PI	SI	SE	FC	ATT	SN	PBC	BI
PU	**.96**										
PEOP	.67	**.95**									
COM	.82	.74	**.98**								
PI	.70	.74	.81	**.98**							
SI	.61	.73	.73	.77	**.96**						
SE	.73	.70	.83	.82	.74	**.95**					
FC	.70	.62	.76	.73	.61	.76	**.95**				
ATT	.77	.79	.88	.82	.80	.80	.70	**.94**			
SN	.71	.75	.79	.86	.84	.82	.70	.84	**.99**		
PBC	.71	.75	.84	.82	.76	.86	.81	.83	.84	**.96**	
BI	.69	.78	.82	.84	.78	.82	.75	.84	.83	.88	**.96**

Diagonal means the square root of Average Variance Extracted

### Structural model

**Fig. 2:**
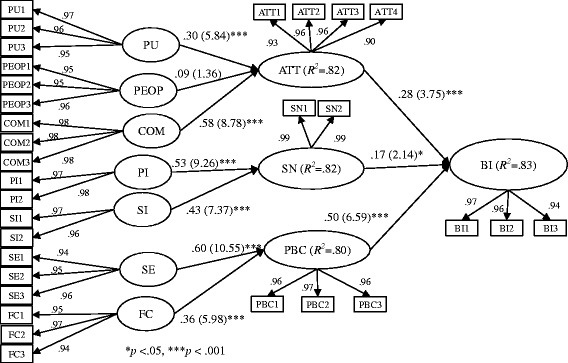
Structural model results with β and t-statistics (in parenthesis)(Adapted with permission. Copyright 1995 INFORMS. Shirley Taylor, Peter A. Todd (1995) Understanding Information Technology Usage: A Test of Competing Models. *Information Systems Research* 6(2):144–176, the Institute for Operations Research and the Management Sciences, 5521 Research Park Drive, Suite 200, Catonsville, Maryland 21228, USA)

After validating the measurement model, we then assessed the hypotheses by examining the structural model. We used the bootstrapping procedure to test the statistical significance of each path coefficient. Figure 2 presents the structural model results with path coefficient and t-statistics. Regarding hypotheses H_1_, H_2_, and H_3_, the results significantly supported only H_1_ and H_3_. That is, attitude was influenced by perceived usefulness (β = .30, *t* = 5.84) and compatibility (β = .58, *t* = 8.78), while perceived ease of protection was not a significant predictor of attitude (β = .09, *t* = 1.36). In terms of hypotheses H_4_ and H_5_, the results revealed significant support for both hypotheses. That is, subjective norm was influenced by peer influence (β = .53, *t* = 9.26) and superior influence (β = .43, *t* = 7.37), and peer influence is the strongest predictor of subjective norm. Further, hypotheses H_6_ and H_7_ were confirmed that both self-efficacy (β = .60, *t* = 10.55) and facilitating conditions (β = .36, *t* = 5.98) positively affect perceived behavioral control. Regarding hypotheses H_8_, H_9_, and H_10_, the results demonstrated that attitude (β = .28, *t* = 3.75), subjective norm (β = .17, *t* = 2.14), and perceived behavioral control (β = .50, *t* = 6.59) contributed to behavioral intention to protect privacy of EMRs. Perceived usefulness and compatibility jointly explained about 82 % of the variance of attitude while peer influence and superior influence roughly accounted for 82 % of the variance of subjective norm. In addition, self-efficacy and facilitating conditions collectively explained about 80 % of the variance of perceived behavioral control. Overall, the model explained about 83 % of the determined variance in the behavioral intention to protect the privacy of EMRs. Further, we adopted the global fit measure (GoF) to validate the overall PLS model and used the formula as $\sqrt{\overline{Average\  Variance\  Extracted(AVE)}}*\left.\overline{R^2}\right)$ to compute the GoF [49]. The average AVE = .93 and average *R* ^*2*^ = .82, resulting in a GoF = .87 demonstrated that our model was valid [49].

## Discussion

### Effects of perceived usefulness, perceived ease of protection, and compatibility on attitude

In agreement with the assertion of DTPB, we found that perceived usefulness (PU) has a positive relationship with attitude [14]. The result is also consistent with prior studies in both healthcare [29, 31, 32, 50] and non-healthcare studies [35, 51], indicating that PU is a stable measure in predicting attitude across differing study contexts. The support of H_1_ demonstrates that an increase in perceived usefulness would strengthen nurses’ attitude toward protecting the privacy of EMRs. In the light of this finding, demonstrating the benefits and importance of protecting EMRs privacy to nurses is essential to foster their positive attitudes. To that end, providing appropriate privacy training may be vital for directing nurses’ beliefs regarding the usefulness of protecting the privacy of EMRs. In that way, nurses may hold more a positive attitude toward protecting EMRs privacy.

The result of failed support for H_2_ is not in line with what the original DTPB postulates. That is, perceived ease of use (PEOU) should have a positive relationship with attitude [14]. However, Lee et al. [52] found that PEOU is an unstable predictor in other contexts. They found only 58 out of 101 studies revealed a significant relationship between perceived ease of use with dependent variables. Although our result did not support the DTPB and related studies in non-healthcare context [35, 38], the results are in line with some prior literature conducted in healthcare context [31, 32]. More specifically, our finding is consistent with literature using physician subjects [32]. Chau and Hu [32] found that physicians might not consider perceived ease of use as an important factor since they can learn technology quickly. But Hung et al. [28] found that perceived ease of use significantly predict physician’s intention to use Medline systems. Our study used nurses as the study subjects and the evidence revealed an insignificant outcome. One possible reason might be that since nurses are familiar with protecting privacy of paper-based medical records, they have no concerns in conducting such protective behavior in digitized medical records. Most nurses should have adequate information literacy after graduation in Taiwan [53] and consequently, nurses might not view ease to protect the privacy as an issue of particular importance.

Regarding H_3_, we validated compatibility to be a significant predictor of nurses’ attitude toward protecting the privacy of EMRs. The result is consistent with the postulation of DTPB [14] and other studies [31, 54, 55]. Tung et al. [56] found that nurses are willing to use electronic logistic information systems only if the system is consistent with their existing values, experiences, and needs. For nurses, the primary value of protection privacy is not different between paper-based and digitized medical records. Further, nurses are used to practicing in a traditional way, that is, documenting/querying patient’s medical records in the nurses’ stations under the regulation of privacy policies. Therefore, most of the procedures for effectively protecting the privacy of EMRs should not be different from protecting paper-based medical records. However, nurses can also query medical records anywhere with proper devices and network connectivity away from nurses’ stations, which may require nurses to undertake a different approach and to possess sufficient IT-related skills for protecting the privacy of EMRs. Consequently, the significant result may imply that hospitals should ensure that any procedures or ITs employed for improving the protection of EMRs privacy should be consistent with nurses’ work practices and designed according to nurses’ experiences and needs.

### Effects of peer influence and superior influence on subjective norm

DTPB decomposed subjective norms into peer influence and superior influence due to the possible incongruence of opinions among various referent groups [14]. While other studies proposed differing referent groups, these differing referent groups consistently exert significant effects on subjective norms. Our study is consistent with DTPB [14] and previous healthcare-related study [29] that decomposed subjective norm into peer influence and superior influence.

Nurses, as a profession, share a common terminology, training, professional culture, and work environments and a tendency towards compliance with organizational norms and expectations [57]. The support of H_4_ may imply that hospitals must equip nurses with adequate ethical knowledge and skills concerning the privacy-protection of EMRs. When nurses understand what kinds of actions are appropriate for accessing EMRs, they may prevent other nurses from committing illegal acts. Further, the support of H_5_ also demonstrates that hospitals should use the knowledge that superior influence reinforces perceptions of subjective norms to train managers, such as head nurses, to supervise other nurses in protecting the privacy of EMRs. Most hospitals have existing privacy policies for protecting EMRs and these policies mandate healthcare professionals, including nurses, adhere. Managers are obligated to ensure that health professionals strictly follow these policies. This practice may explain why superior influence is a significant predictor of subjective norms regarding EMRs privacy-protection.

### Effects of self-efficacy and facilitating conditions on perceived behavioral control

As hypothesis H_6_ postulated, self-efficacy is a significant predictor of perceived behavioral control (PBC). Self-efficacy primarily concerns nurses’ self-confidence in their ability to protect the privacy of EMRs. In Taiwan, nursing schools usually provide lectures or courses concerning patient privacy and information security [53]. Meanwhile, the hospital in this study has acquired the ISO 27001 certification that mandates hospitals hold information-security training programs regularly. Consequently, besides having adequate knowledge regarding EMRs, nurses in our study should be equipped with sufficient privacy-protection knowledge. Further, nurses have adopted ethical codes that address the responsibility toward protecting patient privacy. Thus, nurses are ethically bound to hold all information in confidence [58]. This responsibility may explain why self-efficacy is a significant predictor of perceived behavioral control and the findings are in line with prior literature [14, 28, 29, 37, 59]. This significant finding may indicate that hospitals should provide nurses with sufficient ethical knowledge and IT skills for protecting the privacy of EMRs. We suggest that hospitals could organize continuous training programs regarding ethics, information security concepts/procedures, and IT skills to enhance their capabilities in protecting the privacy of EMRs.

Regarding hypothesis H_7_, facilitating condition is a strong determinant of nurses’ perceived behavioral control toward protecting the privacy of EMRs. Facilitating condition refers to resources such as EMRs related hardware, software, and usability for facilitating privacy-protection behavior that are compatible with existing hardware and software in hospitals. Since the hospital in this study adopted EMRs in 2009, the hospital leadership has integrated all computer hardware, software, and management procedures required for EMRs within the existing Hospital Information Systems (HISs). However, since most hospitals have joined Taiwan’s National Health Insurance Program, EMRs are subject to frequent change in regulations. Hence, nurses may expect that hospitals ensure subsequent changes in regulations that influence EMRs are compatible with existing HISs. This review would mandate the analysis of the requirements carefully whenever the National Health Insurance program implements essential changes that affect EMRs. These recommendations support previous studies [29, 38] and suggest that hospitals should ensure continued compatibility with the EMRs. Thus, nurses should perceive a lesser degree of constraint when protecting the privacy of EMRs.

### Effects of attitude, subjective norm, and perceived behavioral control on behavioral intention to protect privacy of EMRs

H_8_ stated that the attitude of nurses would directly influence their intention to protect the privacy of EMRs. In words, nurses with positive opinions of the need for privacy of EMRs will be more likely to protect the privacy of EMRs, while nurses who simply do not care will be less likely to protect the privacy of EMRs. Attitude is a stable predictor of behavioral intention in previous studies [28–30, 52, 60, 61] and our finding is consistent with results from many previous studies. Based on this finding, we suggest hospitals formulate relevant strategies for cultivating nurses with positive opinions on privacy of EMRs issues. Such strategies could include holding provisional seminars or constant training programs concerning ethics to strengthen nurses’ attitude toward EMRs privacy issue. Further, these training programs could focus on introducing the consequences of violating EMRs privacy policy as the awareness of these issues may lead to a change in nurses’ attitude toward EMRs privacy.

As stated in H_9_, the subjective norm of nurses will positively influence their intention to protect the privacy of EMRs. The results demonstrate that nurses have beliefs that depend on the social norm of referent groups (peer nursing workers and their superiors in our study). These results suggest that social influence plays a critical role in nurse’s privacy-protective intentions and are in line with previous studies [28, 30, 61, 62]. Our findings may suggest that protecting the privacy of EMRs among nurses can be improved by leveraging referent groups or important others that influence nurses’ intention to protect privacy. As Milholland [58] stated, nurses do not want providers or others to inadvertently access patient information. Therefore, nurse managers need to be sensitive to the privacy issues to guide their staffs in protecting patients from unauthorized invasions of privacy. The study produced results that corroborate the findings of original DTPB [14].

H_10_ asserts that the perceived behavioral control of nurses has a significant impact on their intention to protect the privacy of EMRs. In words, when nurses perceive higher control of or feel they are capable of protecting the privacy of EMRs, they are more likely to engage in such protective behavior. The results are consistent with previous studies [28, 30, 31, 37, 52, 61, 62]. Based on the findings, we suggest that hospitals improve nurses’ control perceptions via providing adequate resources and skills to facilitate nurses’ behavioral intention to protect the privacy of EMRs. To achieve the above-mentioned goal, hospitals could provide proper training programs concerning ethics and IT skills to enhance nurses’ perceptions of controllability to protect the privacy of EMRs.

### Limitations and future study directions

Although the results of this study provide educational and pragmatic implications, some limitations create several opportunities for further research. First, our study only measured nurses’ behavioral intention of protecting the privacy of EMRs, which might not be representative of nurses’ actual protecting behavior. Future studies can collect data from nurses’ actual behavior to understand better the relationships among these constructs. Second, we conducted the study using a cross-sectional design, which may lead to a snapshot presentation of the current setting. Thus, additional research would add value to the theoretical development by using longitudinal studies. Third, since our study only targeted nurses in one Taiwanese military hospital, we cannot safely generalize the findings to other hospitals or to other countries. Future research should select the respondents from more representative samples. Finally, although common method bias does not seem to be a serious problem in our study, future study should avoid common method bias before data collection.

## Conclusions

Our study examined a model based on DTPB to explain what motivates nurses to protect patient privacy in EMRs. Using responses collected from 302 nurses practicing in a tertiary care military hospital in Taiwan, we were able to validate the research model empirically in terms of the overall fit and explanatory power as well as the individual causal relationships specified. The model explains about 83 % of the variance in the behavioral intention. Our findings support nine of the 10 proposed hypotheses. The rejected hypothesis showed that perceived ease of protection has no influence on nurses’ attitude toward protecting the privacy of EMRs.

Our study has important implications. As EMRs continue to permeate healthcare industries, hospitals should pay attention to privacy issues as well as the benefits of EMRs. The evidence of our study suggests the development of training interventions that foster nurses’ positive attitudes toward the privacy of EMRs. This training should place emphases on acquainting nurses with the benefits of protecting the privacy of EMRs using protective procedures consistent with nurses’ needs and prior experiences. Additionally, our study shows that subjective norm contributes to nurses’ intention to protect the privacy of EMRs; and that the influence of nurses’ peers and supervisors enhance such perceptions. Finally, perceived behavioral control also contributes to nurses’ privacy-protection intentions about EMRs. Hospitals should augment these control perceptions by providing sufficient skills training and resources.

### Contributions

This study adopted the DTPB as the theoretical underpinning to investigate nurses’ behavioral intention to protect privacy of EMRs. Our findings demonstrate that DTPB provides a strong explanation of nurses’ intention to protect the privacy of EMRs with an R-square statistic for behavioral intention of 83 %. Further, the findings also provide insights for hospital managers to formulate strategies to boost nurses’ intention to protect the privacy of EMRs. Adding to the growing body of literature about privacy-protection among nurses, this study is particularly relevant to hospital managers facing the possibility of unauthorized invasions of patient privacy in EMRs.

## Appendix 1

**Table:** Removed items

Constructs (abbreviation)	Items retained	Items removed	Reason(s) for removing items
Perceived usefulness (PU)	PU1/PU2/PU3	PU4: Overall, protecting EMRs privacy will be advantageous	Two out of three experts considered PU4 is similar to PU1, PU2, and PU3; and suggested removal.

## Abbreviations

ATT: attitude
BI: behavioral intention
COM: compatibility
DTPB: decomposed theory of planned behavior
EHR: electronic health record
EMR: electronic medical record
FC: facilitating conditions
HIPAA: Health Insurance Portability and Accountability Act
HIS: Hospital Information System
HIT: Healthcare information technology
IT: Information technology
PBC: Perceived behavioral control
PEOP: Perceived ease of protection
PEOU: Perceived ease of use
PHR: Personal health record
PI: Peer influence
PU: Perceived usefulness
SE: Self-efficacy
SI: Superior influence
SN: Subjective norm
TPB: Theory of planned behavior
